# Smart Care™ versus respiratory physiotherapy–driven manual weaning for critically ill adult patients: a randomized controlled trial

**DOI:** 10.1186/s13054-015-0978-6

**Published:** 2015-06-11

**Authors:** Corinne Taniguchi, Elivane S. Victor, Talita Pieri, Renata Henn, Carolina Santana, Erica Giovanetti, Cilene Saghabi, Karina Timenetsky, Raquel Caserta Eid, Eliezer Silva, Gustavo F. J. Matos, Guilherme P. P. Schettino, Carmen S. V. Barbas

**Affiliations:** Adult ICU, Hospital Israelita Albert Einstein, Avenida Albert Einstein, 627, 5 andar, São Paulo, SP CEP:05652-900 Brazil; Respiratory ICU, University of São Paulo Medical School, Avenida Dr Eneas de Carvalho Aguiar, 255, 6 andar, São Paulo, CEP: 05403-000 Brazil

## Abstract

**Introduction:**

A recent meta-analysis showed that weaning with SmartCare™ (Dräger, Lübeck, Germany) significantly decreased weaning time in critically ill patients. However, its utility compared with respiratory physiotherapist–protocolized weaning is still a matter of debate. We hypothesized that weaning with SmartCare™ would be as effective as respiratory physiotherapy–driven weaning in critically ill patients.

**Methods:**

Adult critically ill patients mechanically ventilated for more than 24 hours in the adult intensive care unit of the Albert Einstein Hospital, São Paulo, Brazil, were randomly assigned to be weaned either by progressive discontinuation of pressure support ventilation (PSV) with SmartCare™. Demographic data, respiratory function parameters, level of PSV, tidal volume (VT), positive end-expiratory pressure (PEEP), inspired oxygen fraction (FiO_2_), peripheral oxygen saturation (SpO_2_), end-tidal carbon dioxide concentration (EtCO_2_) and airway occlusion pressure at 0.1 second (P_0.1_) were recorded at the beginning of the weaning process and before extubation. Mechanical ventilation time, weaning duration and rate of extubation failure were compared.

**Results:**

Seventy patients were enrolled 35 in each group. There was no difference between the two groups concerning age, sex or diagnosis at study entry. There was no difference in maximal inspiratory pressure, maximal expiratory pressure, forced vital capacity or rapid shallow breathing index at the beginning of the weaning trial. PEEP, VT, FiO_2_, SpO_2_, respiratory rate, EtCO_2_ and P_0.1_ were similar between the two groups, but PSV was not (median: 8 vs. 10 cmH_2_O; *p* =0.007). When the patients were ready for extubation, PSV (8 vs. 5 cmH_2_O; *p* =0.015) and PEEP (8 vs. 5 cmH_2_O; *p* <0.001) were significantly higher in the respiratory physiotherapy–driven weaning group. Total duration of mechanical ventilation (3.5 [2.0–7.3] days vs. 4.1 [2.7-7.1] days; *p* =0.467) and extubation failure (2 vs. 2; *p* =1.00) were similar between the two groups. Weaning duration was shorter in the respiratory physiotherapy–driven weaning group (60 [50–80] minutes vs. 110 [80–130] minutes; *p* <0.001).

**Conclusion:**

A respiratory physiotherapy–driven weaning protocol can decrease weaning time compared with an automatic system, as it takes into account individual weaning difficulties.

**Trial registration:**

Clinicaltrials.gov Identifier: NCT02122016. Date of Registration: 27 August 2013.

## Introduction

A large proportion of intensive care unit (ICU) patients require mechanical ventilation for more than 24 hours [1]. Automatic ventilator control and ventilation modes designed for weaning from mechanical ventilation can be particularly helpful, as 40 % of mechanical ventilation time is spent on this procedure [2]. Owing to the complexity of the decision-making process for weaning, the use of computers has been increasingly favored to ensure that protocols are followed completely and safely [3]. Automated weaning programs were developed to bring better control to this process and to make it faster and more secure [4]. Patients’ information is entered into the program for monitoring, then, through the program’s decision-making process defined by the weaning protocol, the computer suggests changes or maintenance of ventilator parameters [5]. This happens through the direct inclusion of monitored data into the program by the ICU team, whereby the computer defines the action to be taken and the caregivers act accordingly, with the program dictating the protocol. This process can also occur automatically, through the so-called closed-loop ventilation system, when this software is integrated with a mechanical ventilator. The ventilator monitors the patient and, through an internal algorithm, changes ventilation parameters according to the ventilation program in a constant feedback ventilation [5]. In general, ventilation modes in closed loop target the lowest possible ventilatory support needed to properly ventilate the patient, which is why the most popular forms of closed-loop ventilation are those used for weaning from mechanical ventilation. Automated systems use closed-loop control to enable ventilators to perform basic and advanced functions while supporting respiration [6].

A recent meta-analysis showed that weaning with SmartCare™ (Dräger, Lübeck, Germany) significantly decreases weaning time in critically ill patients [7]. SmartCare™ is a unique automated weaning system that measures selected respiratory variables, adapts ventilator output to individual patient needs by operationalizing predetermined algorithms and automatically conducting spontaneous breathing trials (SBTs) when predetermined thresholds are met.

ICU weaning protocols driven by respiratory therapists and/or physiotherapists can also be effective in shortening weaning duration [8, 9]. In the adult ICU of Albert Einstein Hospital, São Paulo, Brazil, there is a weaning protocol [10] that consists of a daily assessment for the possibility of an SBT. The ICU SBT is performed in pressure support ventilation (PSV), which allows for better patient monitoring, a greater control of inspired oxygen fraction (FiO_2_) and the maintenance of positive end-expiratory pressure (PEEP). The SBT is performed as soon as the daily assessment indicates that a weaning trial is feasible. If the SBT is successful, the patient is extubated. Otherwise, patients return to the previous ventilation mode and another daily assessment of readiness for weaning is performed the next day (24-hour interval). If it succeeds, another SBT is attempted [10].

We hypothesized that the weaning strategy with SmartCare™ ventilatory mode would be as effective as our respiratory physiotherapy–driven weaning protocol in the ICU patients receiving invasive mechanical ventilation for more than 24 hours. Therefore, the objective of this study was to compare SmartCare™ ventilatory mode with our validated respiratory physiotherapy–driven weaning protocol [10] in critically ill patients receiving invasive mechanical ventilation for more than 24 hours. We sought to evaluate the following:

1. Primary outcome: duration of weaning or weaning time (from randomization to extubation)
2. Secondary outcomes:
  - Rate of extubation failure (the need to return to invasive mechanical ventilation within 48 hours of extubation)
  - Mechanical ventilation duration or mechanical ventilation time (from intubation to extubation)
  - Physiologic measurements: respiratory rate (f), tidal volume (VT), rapid shallow breathing index (RSBI; calculated as frequency of breaths [respiratory rate] divided by tidal volume in liters [f/VT {L}]), forced vital capacity (FVC), level of PSV, PEEP, FiO_2_, end-tidal carbon dioxide concentration (EtCO_2_) and airway occlusion pressure at 0.1 second (P_0.1_) at the beginning of weaning and before extubation

## Methods

A randomized, unblinded, prospective, controlled clinical trial was carried out in 70 consecutive adult critically ill patients who were intubated and mechanically ventilated for more than 24 hours in the adult ICU of Albert Einstein Hospital, São Paulo, Brazil. The study was approved by the ethics committee of Albert Einstein Hospital (CEP: 1506) and signed at “Plataforma Brasil” (SGPP 1260-10). Signed informed consent was obtained from each patient or the patient’s next of kin. The study was registered at ClinicalTrials.gov on 27 August 2013 (NCT02122016).

The inclusion criteria were adult patients on mechanical ventilation for more than 24 hours who were able to initiate spontaneous inspiratory effort, presenting spontaneous eye opening and response to stimuli with minimal level of sedation, and in whom the cause that led or contributed to the need of mechanical ventilation was resolved. The patients had to have an FiO_2_ less than 0.4, peripheral oxygen saturation (SpO_2_) greater than 93 % and PEEP less than 10 cmH_2_O. They had to be hemodynamically stable as expressed by good tissue perfusion, independence from or low doses of vasopressors (dosage of vasoactive drug less than 0.05 μg/kg/min) and absence of decompensated coronary insufficiency or arrhythmias with hemodynamic repercussion. The exclusion criteria were presence of tracheostomy, neurologic damage with limited prognosis (after cardiac arrest or central neurologic damage) or Glasgow Coma Scale score less than 10.

Subsequently, the patients screened as suitable for weaning were subjected to assessment of the following:

1. Maximal inspiratory pressure (Pimax) measurement with a manometer (Commercial Medical Electronics, Tulsa, OK, USA) and a unidirectional valve (Hudson RCI; Teleflex, Morrisville, NC, USA) connected to the tube (Hudson RCI) and with use of a bacterial filter (Gibeck ISO-Gard; Teleflex) (Measurements were based on functional residual capacity.)
2. Maximal expiratory pressure (Pemax) measurement with a manometer based on total lung capacity
3. Spontaneous VT and FVC measurements with a spirometer (Wright spirometer, British model) plugged into the airway through a bacterial filter (ISO-Gard) (We measured initially minute ventilation in liters per minute and respiratory rate (f) for 1 minute to calculate VT. Next, to measure FVC, the respiratory physiotherapist ensured that the patient breathed in as deeply as possible and then asked the patient to forcibly blast the breath out as fast and as long as possible.)
4. RSBI (We directly connected the Wright ventilometer [British model] to the endotracheal tube to measure the patient’s spontaneous VT and the respiratory rate (f) for a duration of 1 minute. The index was obtained by calculating f/VT [L].)

**Fig. 1:**
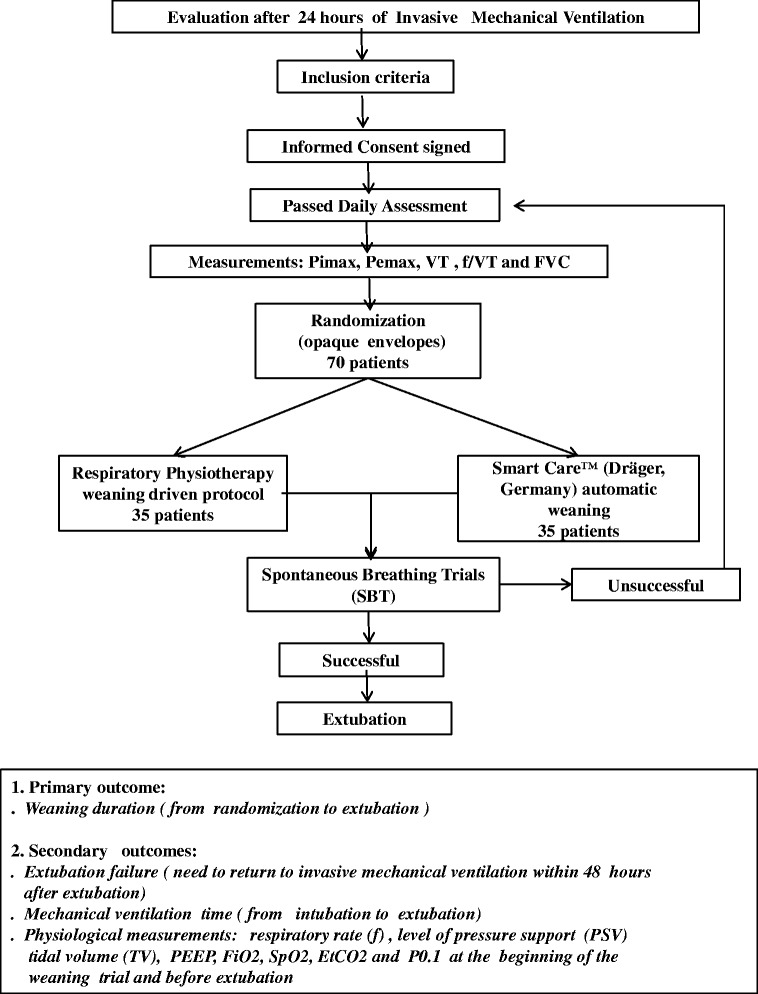
Study flowchart. EtCO_2_, End-tidal carbon dioxide concentration; f/VT, Frequency of breaths (respiratory rate) divided by tidal volume; FiO_2_, Inspired oxygen fraction; FVC, Forced vital capacity; P_0.1_, Airway occlusion pressure at 0.1 second; PEEP, Positive end-expiratory pressure; Pemax, Maximal expiratory pressure; Pimax, Maximal inspiratory pressure; PSV, Pressure support ventilation; SpO_2_, Peripheral oxygen saturation; VT, Tidal volume

After the measurements were performed, the patients were randomized by drawing folded slips of paper from a large, opaque envelope. Each slip of paper provided an identification number that assigned the patient to one of the two weaning modes (Fig. 1): (1) the respiratory physiotherapy–driven group or (2) the weaning with SmartCare™ group.

### Respiratory physiotherapy–driven weaning group

The randomized patients assigned to the respiratory physiotherapy–driven weaning group were ventilated on continuous positive airway pressure (CPAP)/PSV mode (EVITA XL; Dräger) using PSV mode. All the patients were subjected to an SBT, which consists of an integrated assessment of the patients while they breathe spontaneously through PSV of 5–7 cmH_2_O and PEEP of 5 cmH_2_O. The SBT in the PSV mode is carried out for a minimum of 30 minutes and a maximum of 2 hours. Respiratory rate, level of PSV, VT, PEEP, FiO_2_, SpO_2_, EtCO_2_ and P_0.1_ were recorded at the beginning of the weaning process and before extubation. Patients who failed the SBT were returned to the PSV ventilatory mode that was titrated to a minimum level to ensure patient comfort: f <28 breaths/min and VT >5 ml/kg of predicted body weight. SBT failure was defined as tachycardia (heart rate >140 beats/min); increased respiratory rate (f >35 breaths/min); SpO_2_ <90 %; systolic blood pressure >180 mmHg or <90 mmHg; and signs or symptoms of agitation, sweating or altered level of consciousness. After the patients who failed an SBT were returned to the PSV ventilatory mode, they were then reevaluated to identify the causes of the failure. After 24 hours, the patients were reevaluated again, and, if they passed the daily assessment for eligibility to wean, a new SBT was performed. The duration of weaning (from randomization to extubation), the rate of extubation failure (need to return to invasive mechanical ventilation within 48 hours after extubation) and mechanical ventilation time (from intubation to extubation) were recorded.

### Automatic weaning group (SmartCare™ group)

The patients were ventilated on CPAP/PSV mode (EVITA XL) using PSV mode. The icon SmartCare™ was triggered, and a set of relevant information was adjusted: patient’s weight, type of tracheal prosthesis, whether the patient had neurological disease or chronic obstructive pulmonary disease and whether the mode was to work throughout the night. The apnea parameters were then set for apnea ventilation: f of 15 breaths per minute, VT of 6 ml/kg of predicted body weight and flow trigger sensitivity of 2 L/min. After these adjustments, the SmartCare™ mode was then initiated. The ventilator automatically adjusted pressure support at the minimum level while keeping the patient within a comfort zone. If the patient’s condition developed satisfactorily, the ventilator attempted an SBT and sounded an alarm when the patient was ready for extubation. As soon as the warning alarm illuminated and sounded, it was quickly detected by the bedside physiotherapy team, who immediately extubated the patient. Respiratory rate, level of PSV, VT, PEEP, FiO_2_, SpO_2_, EtCO_2_ and P0.1 were recorded at the beginning of the weaning process and before extubation. Weaning duration, extubation failure rate and mechanical ventilation duration were also recorded.

### Statistical data analysis

Absolute frequencies and percentages of categorical variables were described for each group. Comparisons between groups were performed by χ^2^ or Fisher’s exact tests. Normality in the distribution of variables within each group was tested with the Shapiro-Wilk test, and homogeneity of variances was assessed with Levene’s test. Normally distributed variables were described by their mean and standard deviation and compared using Student’s *t* tests. Otherwise, medians and interquartile ranges (IQRs) were used, and the Mann–Whitney U test was used for comparisons. A Bonferroni correction for multiple comparisons was applied if necessary. Analyses were performed with IBM SPSS version 17.0 software (IBM, Armonk, NY, USA). *p* values less than 0.05 were considered statistically significant.

## Results

### Patients’ characteristics

A total of 70 consecutive patients were enrolled, 35 in each weaning protocol group. Mean age, gender distribution and patient diagnoses were similar between the two groups (Table 1). Diagnoses at patient ICU admission were predominantly respiratory, hepatic, gastrointestinal and infectious. They did not differ between the two groups.

**Table 1:** Characteristics of ICU patients under the two weaning protocols: respiratory physiotherapy–driven weaning and automatic weaning (SmartCare™ mode)

		Weaning mode				*p* Value
		Respiratory physiotherapy–driven (*n* =35)		SmartCare™ (*n* =35)		
		*n*	%	*n*	%	
Diagnosis at ICU admission						
	Cardiac	2	5.7 %	1	2.9 %	0.722
	Gastrointestinal	7	20.0 %	6	17.1 %	
	Hepatic	7	20.0 %	6	17.1 %	
	Infection	3	8.6 %	8	22.9 %	
	Neurologic	2	5.7 %	2	5.7 %	
	Oncologic	0	0.0 %	1	2,9 %	
	Orthopedic	1	2.9 %	0	0.0 %	
	Respiratory	13	37.1 %	11	31.4 %	
	Total	35	100.0 %	35	100.0 %	
Age, yr		66 ± 18 (20–93)		62 ± 19 (33–97)		0.388
Sex						
	Male	17 (49 %)		22 (63 %)		0.336
	Female	18 (51 %)		13 (37 %)		

When we compared the two weaning groups, we found no significant difference in the Pimax (*p* =0.270) and Pemax (*p* =0.058) measured before the SBT. There was also no significant difference between the two groups concerning the FVC or the RSBI (Table 2).

**Table 2:** Maximum inspiratory and expiratory pressures, rapid shallow breathing capacity and forced vital capacity before weaning start among ICU patients

	Weaning mode		*p* Value
	Respiratory physiotherapy driven, median (IQR)	Smart Care™, median (IQR) group	
f/VT	40 (26–71)	35 (24–56)	0.404
FVC	975 (900–1550)	1200 (900–1900)	0.683
Pimax	−40 (35–50)	−45 (40–50)	0.270
Pemax	40 (22–45)	40 (30–60)	0.058

*Abbreviations: f/VT* respiratory rate divided by tidal volume in liters; *FVC* forced vital capacity in milliliters, *P* *e* *max* maximum expiratory pressure (cmH_2_O), *P* *i* *max* maximum inspiratory pressure (cmH_2_O)

There was no difference in the need for reintubation between groups (two patients from each group required reintubation; *p* =1.00) or in the frequency of mechanical ventilator malfunctions between the ventilation modes (*p* =0.239). However, three technical hitches were observed in the automatic mode. Two difficulties in the calibration of EtCO_2_ coupled to the ventilator, and one difficulty during the decrease of PSV caused by psychomotor agitation of the patient led to tachypnea.

There were more clinical complications during the weaning in the SmartCare™ group (*p* <0.001) (Table 3), which were not related to the ventilator mode itself. Of the two complications in the respiratory physiotherapy–driven weaning group, one was the result of a delay in performing a cuff leak test owing to psychomotor agitation and delirium, and the other was associated with a patient’s episode of arrhythmia during the weaning process, followed by medication before the process could continue. There were six clinical complications in the SmartCare™ group. One involved a positive cuff leak test that indicated upper-airway edema which needed to be treated with corticosteroids. One patient had temporary hemodynamic instability that had to be treated before extubation. One patient had gastrointestinal bleeding. One patient had nausea impairing the extubation. One was a case of difficult airway that the attending physician determined required extubation by bronchoscopy. One was a patient who needed insertion of a femoral catheter (for dialytic procedure) just after the weaning process was started.

**Table 3:** Reintubation rate, ventilator malfunction and clinical complications in the two weaning protocols

	Weaning mode				*p* Value
	Respiratory physiotherapy–driven weaning		SmartCare™ group		
	*n*	%	*n*	%	
Reintubation rate	2	5.7 %	2	5.7 %	1.00
Ventilator related malfunction	0	0.0 %	3	8.6 %	0.239
Clinical complications not related to the ventilatory mode itself	2	5.7 %	6	22.9 %	0.001

### Similar ventilation time, but shorter weaning duration, associated with respiratory physiotherapy–driven weaning protocol

There was no difference between the two groups at the beginning of the weaning trial in FVC (*p* =0.683), f/VT (L) (*p* =0.404), PEEP (*p* =0.944), VT (*p* =0.509), FiO_2_ (*p* =0.499), SpO_2_ (*p* =0.774), *f* (*p* =0.947), EtCO_2_ (*p* =0.422) or P_0.1_ (*p* =0.201), except that PSV that was lower in the respiratory physiotherapy–driven weaning group (8 [IQR: 7–10] cmH_2_O vs. 10 [8–12] cmH_2_O; *p* =0.007). When the patients were ready for extubation, PSV was significantly higher in the respiratory physiotherapy–driven weaning group (8 [IQR: 7–8] cmH_2_O vs. 5 [IQR: 5–8] cmH_2_O; *p* =0.015), as was PEEP (8 [IQR: 7–8] cmH_2_O vs. 5 [IQR: 5–5] cmH_2_O; *p* <0.001), indicating that the decision to extubate was more rigidly controlled in the SmartCare™ automatic group (Table 4). Total duration of mechanical ventilation was similar in both groups (*p* =0.467). However, weaning duration and weaning time in the respiratory physiotherapy–driven weaning group were significantly shorter (60 [50–80] minutes vs. 110 [80–130] minutes; *p* <0.001) (Table 5).

**Table 4:** Respiratory parameters at the weaning start and end

	Start of weaning			End of weaning		
Weaning mode	Respiratory therapy–driven weaning	SmartCare™ weaning		Respiratory therapy–driven weaning	SmartCare™ weaning	
	Median (IQR)	Median (IQR)	*p* Value	Median (IQR)	Median (IQR)	*p* Value
VT (ml)	491 (350–611)	515 (413–612)	0.509	500 (420–600)	500 (450–630)	0.535
*f* (breaths/min)	20 (16–24)	20 (17–24)	0.947	17 (15–20)	18 (17–20)	0.354
PSV (cmH_2_O)	8 (7–10)	10 (8–12)	0.007	8 (7–8)	5 (5–8)	0.015^a^
PEEP (cmH_2_O)	8 (8–10)	8 (8–10)	0.944	8 (7–8)	5 (5–5)	<0.001^b^
FiO_2_ (%)	0.30 (0.25–0.30)	0.30 (0.25–0.30)	0.499	0.30 (0.25–0.30)	0.30 (0.25–0.30)	0.572
SpO_2_ (%)	99 (98–100)	99 (98–100)	0.774	99 (98–100)	99 (98–99)	0.617
P_0.1_ (cmH_2_O)	2.5 (0.7–4.2)	1.3 (0.7–2.5)	0.201	2.8 (0.6–4.1)	1.6 (1.2–2.4)	0.201
EtCO_2_ Mean (SD)	35.7 (7.4)	33.6 (8.2)	0.422	35.4 (7.2)	34.9 (6.6)	0.826

*Abbreviations: EtCO*_*2*_ end-tidal expiratory carbon dioxide, *f* respiratory rate, *F* *i* *O*_*2*_ inspired fraction of oxygen, *IQR* interquartile range, *P*_*0.1*_ airway occlusion pressure at the first millisecond, *PEEP* positive end-expiratory pressure, *PSV* pressure support ventilation, *SD* standard deviation, *SpO*_*2*_ peripheral oxygen saturation, *VT* tidal volume

^a^ *p* <0.05

^b^ *p* <0.01

**Table 5:** Mechanical ventilation time and weaning duration in the two weaning protocols

	Weaning mode				*p* Value
	Respiratory physiotherapy–driven		SmartCare™ group		
Mechanical ventilation time (days), median (IQR)	3.5	(2.0–7.3)	4.1	(2.7–7.1)	0.467
Weaning duration (min), median (IQR)	60.0	(50.0–80.0)	110.0	(80.0–130)	<0.001

*Abbreviation: IQR* interquartile range

## Discussion

The results of this study demonstrate that weaning time using SmartCare™ was longer than that of the respiratory physiotherapy–driven protocol. A recent meta-analysis [7] involving 496 participants (7 trials of time from randomization to extubation) [11–17] showed that weaning time using SmartCare™ was decreased compared with non-automated weaning (MD −2.68 days; 95 % confidence interval [CI] −3.99 to −1.37; *p* value <0.0001) in the presence of substantial heterogeneity (*I*^2^ =68 %; *p* =0.005). One possible explanation for the unexpected result of this trial that showed an increased weaning time in the SmartCare™ group is that weaning time in the meta-analysis was defined as the time from randomization to extubation (as determined by the authors) [7]. Weaning time as defined by authors can vary among studies as follows: (1) from the initiation of spontaneous breathing (CPAP), (2) from the initiation of an assisted mode of ventilation (e.g., pressure support, volume-assisted, Proportional Assist Ventilation Plus [PAV+; Covidien, Boulder, CO, USA], adaptive support ventilation, Automatic tube compensation), or (3) as the patient is ready to undergo an SBT. In this study, we analyzed critically ill patients ventilated for more than 24 hours who successfully passed through a daily assessment of weaning evaluation, after which the SBT and extubation were quicker in the respiratory physiotherapy–driven protocol. This finding may be attributable to more efficient management of the intercurrences and individual variability, as there were human brains behind the decisions. Another explanation for the prolonged weaning with SmartCare™ is that more clinical complications not related to the ventilatory mode itself occurred in this group, which may have delayed the patients’ extubation procedures.

If the start of a weaning trial is considered to be when the patient commences assisted ventilation, the weaning time would be increased compared with a patient who successfully passed a complete daily assessment for weaning evaluation. In terms of weaning duration, it took, on average, 60 and 110 minutes for the respiratory physiotherapy–driven weaning and SmartCare™ mode, respectively. Although this difference was significant, both values are within the ideal interval established in the literature for the performance of SBTs (30–120 [18-20] minutes). In a recent meta-analysis [7], the pooled results of 7 studies (496 patients) [11–17] showed that weaning time for SmartCare™ weaning groups was 4.57 (3.77) days vs. 7.56 (10.52) in non-automated groups. This much greater weaning time derived from the meta-analysis vs. minutes observed in the present study should represent a less sick ICU population or that our complete daily assessment for weaning is more complete and better selected the readiness for weaning and extubation. The low reintubation rate for this study (i.e., two cases in each arm [5.7 %]) reinforces the hypothesis that our patients recovered well from the respiratory failure and were in fact able to be extubated from the ventilator after the SBTs.

The results of this study show that total invasive mechanical ventilation times were similar for both weaning modes; average mechanical ventilation duration was approximately 4 days in both weaning modes, a shorter time than previously reported values [8, 9]. In a recent meta-analysis, authors reported pooled data of 7 trials involving 520 participants and showed a significant reduction in total duration of mechanical ventilation of 1.8 days favoring SmartCare™ (MD: −1.68 days; 95 % CI: −3.33 to −0.03; *p* =0.05) with substantial heterogeneity (*I*^2^ =53 %; *p* =0.05) [11, 17–21]. The mean mechanical ventilation time (pooled data from 7 studies, 520 participants [11–17, 21]) was 8.34 (5.88) days for SmartCare™ versus 10.48 (8.25) days for non-automated weaning, showing that the condition of the patients in these seven polled studies was more severe than that of the patients in the present study.

We believe that the efficacy of this respiratory physiotherapy–driven weaning protocol derives from the structure and composition of the ICU professionals in our institution. The adult ICU of Albert Einstein Hospital has physiotherapists (physiotherapists in Brazil also act as respiratory therapists) 24 hours per day, with one physiotherapist for every six to eight patients. These therapists are highly trained in weaning protocols of mechanical ventilation [8, 10, 22, 23]. The physiotherapist is able to watch patients closely, quickly identifying patients who are prepared for SBT through a daily assessment [8, 22, 24] in accordance with the Institute for Healthcare Improvement recommendations for the quality of care in ICUs. There are daily multidisciplinary rounds with clear goals for the assessment of the patients. Once all the patients’ goals have been met, there is a team discussion about each patient’s progress, which also speeds up the process of weaning. Therefore, it is possible that automatic modes could be faster in a service with fewer physiotherapists.

It is worth noting the occurrence of no malfunction events related to the ventilator in the respiratory physiotherapy–driven weaning group, as well as three in the automatic mode, two of which were related to a malfunction of the capnography that prevented PSV from being decreased. After further inspection, these problems were found to be related to the EtCO_2_ cable that connects the ventilator to the cuvette where EtCO_2_ rate is measured. Following cable replacement, this problem was solved. Although previous studies with SmartCare™ have not reported this problem [3, 25–27], the observation of this problem in two of six units used indicates that manufacturers should ensure that EtCO_2_ cables are properly adapted. There was also one case where psychomotor agitation and tachypnea led to an unnecessary increase in PSV in the automatic weaning mode. A similar problem was previously reported [25] where a child was not weaned in automatic mode, owing to pain and tachypnea. Although the influence of respiratory rate for PSV adjustment was less frequent with SmartCare™ mode than with previously studied automatic systems [23], these findings indicate that automatic weaning may not be ideal for patients with tachypnea as a result of pain, fever or delirium, even when more parameters in addition to respiratory rate are monitored.

We acknowledge that there are some limitations of this study. First, the study included only 70 patients (35 in each mode)—a small sample size, but sufficient for the detection of significant differences in weaning duration. Second, the study was conducted among adults; hence, it is not possible to generalize our findings with regard to other age groups. Further research should prove fruitful in this respect. Finally, we tested the SmartCare™ system after the patients successfully passed a complete daily assessment for weaning readiness, and this fact might have decreased the effectiveness of SmartCare™ performance during the weaning phase of invasive mechanical ventilation.

## Conclusions

A respiratory physiotherapy–driven weaning protocol can decrease weaning time compared with an automatic system, as it takes into account individual weaning difficulties. However, both modes have been shown to be safe in terms of the frequency of weaning failure and weaning completion, leading to the same invasive mechanical ventilation duration.

## Key messages

- A complete daily assessment of readiness for weaning in critically ill patients invasively mechanically ventilated for more than 24 hours can help clinicians choose a better modality for patients able to perform SBTs.
- A respiratory physiotherapy–driven weaning protocol can decrease weaning time compared with the SmartCare™ mode, as it takes into account individual weaning difficulties.
- SmartCare™-driven weaning compared with a respiratory physiotherapy–driven weaning led to similar mechanical ventilation duration, weaning completion and failure.

## Abbreviations

CI: Confidence interval
CPAP: Continuous positive airway pressure
EtCO_2_: End-tidal carbon dioxide concentration
f: Respiratory rate
f/VT (L): Frequency of breaths (respiratory rate) divided by tidal volume in liters
FiO_2_: Inspired oxygen fraction
FVC: Forced vital capacity
ICU: Intensive care unit
IQR: Interquartile range
MD: Mean difference
P_0.1_: Airway occlusion pressure at 0.1 second
PAV+: Proportional Assist Ventilation Plus
PEEP: Positive end-expiratory pressure
Pemax: Maximal expiratory pressure
Pimax: Maximal inspiratory pressure
PSV: Pressure support ventilation
RSBI: Rapid shallow breathing index
SBT: Spontaneous breathing trial
SpO_2_: Peripheral oxygen saturation
VT: Tidal volume
